# Reply to: *Delirium* and sleep quality in the intensive care unit: the role of melatonin

**DOI:** 10.62675/2965-2774.20250149

**Published:** 2025-07-15

**Authors:** Rodrigo Bernardo Serafim, Pedro Henrique Rigotti Soares

**Affiliations:** 1 Instituto D’Or de Ensino e Pesquisa Rio de Janeiro RJ Brazil Instituto D’Or de Ensino e Pesquisa - Rio de Janeiro (RJ), Brazil.; 2 Universidade Federal do Rio de Janeiro Department of Internal Medicine Rio de Janeiro RJ Brazil Department of Internal Medicine, Universidade Federal do Rio de Janeiro - Rio de Janeiro (RJ), Brazil.; 3 Grupo Hospital Conceição Hospital Nossa Senhora da Conceição Adult Intensive Care Unit Porto Alegre RS Brazil Adult Intensive Care Unit, Hospital Nossa Senhora da Conceição, Grupo Hospital Conceição - Porto Alegre (RS), Brazil.; 4 Universidade Federal do Rio Grande do Sul Hospital de Clínicas de Porto Alegre Adult Intensive Care Unit Porto Alegre RS Brazil Adult Intensive Care Unit, Hospital de Clínicas de Porto Alegre, Universidade Federal do Rio Grande do Sul - Porto Alegre (RS), Brazil.

**To the Editor,**

We read with interest the correspondence by Finsterer et al. and would like to offer a few comments.^(1)^ Sleep disruption and *delirium* in the intensive care unit (ICU) result from a complex interplay of individual, environmental, and pharmacological factors. The influence of ICU type, genetic predisposition, psychiatric history, and internal stressors is indeed relevant and contributes to this multifactorial syndrome.^(2,3)^

While acknowledging these numerous contributors, it is important to recognize that many are not modifiable. In addition, several interventions commonly associated with *delirium*, such as sedation, mechanical ventilation, and invasive monitoring, are often indispensable for the appropriate management of critical illness. In daily ICU practice, clinical focus frequently shifts toward symptom control and the implementation of feasible, low-risk strategies, particularly when high-quality evidence is limited. Within this context, sleep hygiene optimization and melatonin use may serve as adjunctive tools rather than substitutes for comprehensive *delirium* prevention protocols.^(4)^

Nonpharmacological interventions remain the cornerstone of *delirium* prevention and management in the ICU. Addressing modifiable risk factors is essential, including medication reconciliation, immobility, sleep disruption, and sensory deprivation. Furthermore, proper triage and the allocation of truly high-risk patients to intensive care while avoiding inappropriate ICU admissions are also key strategies for *delirium* prevention.^(5,6)^

Melatonin is not recommended as a direct treatment for *delirium*, but it may aid in restoring circadian rhythms and improving sleep quality, which could indirectly reduce the risk of *delirium*.^(7,8)^ Given the limited number of pharmacologic options with proven efficacy in the ICU setting, melatonin remains a biologically plausible and safe candidate that warrants further investigation.^(3,7)^

Finally, we agree that *delirium* requires prompt recognition and, in selected cases, psychiatric consultation. Nonetheless, early and multimodal prevention must be a shared responsibility of the multidisciplinary team. Intensivists should be able to promptly identify, treat, and address underlying causes in critically ill patients. Therefore, systematic screening using validated tools such as Confusion Assessment Method for the Intensive Care Unit (CAM-ICU) or Intensive Care *Delirium* Screening Checklist (ICDSC), ideally integrated into daily clinical assessments, is essential for timely diagnosis and management.^(3)^
